# Purification of Spent Hop Cone (*Humulus lupulus* L.) Extract with Xanthohumol Using Mesoporous Superparamagnetic Iron Oxide Nanoparticles

**DOI:** 10.3390/antiox14030314

**Published:** 2025-03-05

**Authors:** Natalia Żuk, Sylwia Pasieczna-Patkowska, Ewelina Grabias-Blicharz, Magdalena Pizoń, Jolanta Flieger

**Affiliations:** 1 Department of Analytical Chemistry, Medical University of Lublin, Chodźki 4A, 20-093 Lublin, Poland; natalia.zuk@umlub.pl (N.Ż.); ewelinagrabias@gmail.com (E.G.-B.); magdalena.pizon@umlub.pl (M.P.); 2 Faculty of Chemistry, Department of Chemical Technology, Maria Curie-Skłodowska University, Pl. Maria Curie-Skłodowskiej 3, 20-031 Lublin, Poland; sylwia.pasieczna-patkowska@mail.umcs.pl

**Keywords:** xanthohumol, magnetic dispersion extraction, iron oxide nanoparticles, spent hops

## Abstract

(1) Background: Over 90% of hop crops are currently used in beer production, with a small part used in the cosmetics and pharmaceutical industries. Spent hops as a waste product contain one of the strongest antioxidants, xanthohumol. The aim of the study was to purify spent hop extracts by magnetic dispersive extraction using iron oxide nanoparticles (IONP) to obtain pure xanthohumol; (2) Methods: The extract from the waste product obtained after supercritical carbon dioxide extraction of hops was prepared by ultrasound-assisted extraction utilizing different solvents, i.e., ethyl acetate, propanol, acetone, 80% methanol, ethyl acetate-methanol (1:1, *v*/*v*), and propanol-methanol (1:1, *v*/*v*). The hydrodynamic diameters and zeta potential of IONPs before and after incubation were measured by dynamic light scattering (DLS). The extracts were analyzed by reversed-phase high-performance liquid chromatography (HPLC). Isolated xanthohumol was identified based on the DAD spectrum in the range of 200–600 nm and by Fourier transform infrared spectroscopy/attenuated total reflectance (FT-IR/ATR); The antioxidant activity of extracts before and after incubation with IONPs was assessed using SNPAC (Silver Nanoparticle Antioxidant Capacity), DPPH (2,2-diphenyl-1-picrylhydrazyl radical), and FRAP (Ferric Reducing Antioxidant Power) assays, as well as total phenolic content (TPC) and total flavonoid content (TFC). (3) Results: The amount of added IONPs, the kind of solvent, and the contact time of the extract with nanoparticles were optimized. We found that 80% MeOH extract after incubation with IONPs (865 µg IONPs/g of spent hops) at room temperature for 48 h contains 74.61% of initial xanthohumol content, providing a final xanthohumol concentration of 43 µg mL^−1^. (4) Conclusions: The proposed method of magnetic dispersive extraction using IONPs allows for the purification of spent hops extract and obtaining a pure product, namely xanthohumol, with a wide potential for practical applications in medicine, pharmacy, cosmetics, and agriculture. This is clear evidence of the usefulness of IONP as an effective sorbent. The method allows the use of residues from the brewing industry, i.e., the biomass of used hop cones to obtain a valuable substance.

## 1. Introduction

Common hop (*Humulus Lupulus* L.) is a plant from the hemp family (Cannabaceae Endl.). The female fruiting bodies of this plant, the so-called hop cones, are used mainly in brewing. The most important components of this raw material are resins (hard and soft), essential oils, tannins, polyphenols, and other substances, such as proteins, monosaccharides, amino acids, fats, and waxes. The traditional division of resins into hard and soft fractions is related to their solubility. Hard resins, which are oxidized soft resins, dissolve in cold methanol and diethyl ether but are insoluble in hexane and low-boiling paraffin hydrocarbons [1]. Among the polyphenolic compounds constituting the components of hard resins, the so-called bitter compounds prenylflavonoids can be distinguished, i.e., xanthohumol, demethylxanthohumol, 6-prenylnaringenin, and 8-prenylnaringenin [2,3,4]. Hop bitter compounds exhibit strong antioxidant, antibacterial, antifungal, antiviral, and even anticancer effects [5,6,7,8]. Due to the good health-promoting effects of xanthohumol, methods to obtain high-purity xanthohumol have become a research hotspot in recent years. The main method of obtaining this compound is isolation from natural raw materials or chemical synthesis. Xanthohumol was first isolated by Power et al. in 1913. According to the SciFinder database, the synthesis of xanthohumol was developed at the beginning of the 21st century. The first methods of synthesis from fluoroacetophenone, after six steps, achieved a low yield of about 10% [9]. In the case of chemical synthesis, the selection of the appropriate substrate is important. For example, the patent (WO2022233428A1) describes a method of synthesizing xanthohumol from naringenin. In each case, the synthesis products require purification of the post-reaction mixture by chromatographic methods or crystallization. Biogenic synthesis methods are also known. Yang et al. designed the biosynthesis of xanthohumol using yeast cells Saccharomyces cerevisiae [10]. The main natural source of xanthohumol is *Humulus lupulus* L. The content of xanthohumol in hop raw material depends on the variety and can range from 2% of the dry weight of the raw material [3]. Polish hop varieties usually contain up to 1% xanthohumol [6]. A good source of xanthohumol is post-extraction hops, which are waste from the production of hop extract used by brewers to produce beer. It is estimated that post-extraction hops, the so-called hop waste, contain up to 1% xanthohumol, depending on the hop species. The use of waste products is currently a requirement of sustainable development friendly to the environment. The recovery of substances with antioxidant character can be useful for a wide range of applications in the food, cosmetic, and pharmaceutical industries.

There are many techniques for extracting bioactive compounds from plant materials, starting from classical extraction in the Soxhlet apparatus, maceration, and hydrodistillation. An important stage in the extraction processes of bioactive substances is the optimization of independent variables, i.e., the choice of solvent, extraction pressure, temperature, time, and solid–liquid ratio [11,12,13,14], influencing its efficiency. The use of organic solvents such as hexane, ethanol, and methylene chloride for the extraction of xanthohumol from hop extract is not selective, and other components are extracted, mainly polyphenols, as well as hard resins and waxes. Liquid CO_2_ extraction is characterized by high energy consumption, high costs, and high resistance to mass transfer of the dissolved substance in the liquid, while supercritical CO_2_ extraction of hop extract eliminates hard resin and wax, and although it is environmentally friendly, the quality of the product is still not very satisfactory.

There is not much work on the use of spent hops to obtain satisfactory xanthohumol concentrations. Grudniewska and Popłoński [15] used deep eutectic solvents (DES) composed of choline chloride and propylene glycol (1:2 mol/mol) with 5 wt% water to isolate xanthohumol from spent hops. In the next steps, this method requires the addition of water as an antisolvent and further extraction using methanol. Xanthohumol recovery from spent hops achieved 2.30 mg/g.

Xanthohumol can be isolated from hop cone extracts using Sephadex LH-20 gel chromatography, semi-preparative HPLC on a C18 column [16], or high-speed counter-current chromatography [17]. Other methods for purifying xanthohumol are also described in the literature, including solvent extraction using chloroform and dichloromethane, adsorption on diatomaceous earth, precipitation as a salt, and recrystallization, which enables the production of “Xantho-Pure” with a purity of over 90% [18,19,20].

Another method was proposed by Biendl [19] utilizing the extraction of hops or spent hops with ethanol and fractionation of the resulting extract with supercritical carbon dioxide. It should be emphasized that the xanthohumol should be further isolated by selective adsorption on polyvinylpolypyrrolidone (PVPP). The final product contains a comprehensive range of hop prenylflavonoids, with xanthohumol representing a proportion of between 9 and 80%. In recent years, many patents have also been published for the production of xanthohumol by solvent extraction methods (DE19939350A1, EP1424 385B1, CN101811950), sorption on various sorbents such as diatomaceous earth (CN 101440029) or various adsorption resins (CN101433592), polyamides such as polyvinylpyrrolidone (DE2223698A1), and precipitation methods (EP2187899B1, US 2009/258094, US 11/790,365, DE2223698A1). It should be emphasized that these methods are usually multi-stage, with different, often low efficiencies, and they use organic solvents, which are not beneficial to the environment when produced on an industrial scale. In addition, when the temperature is increased during these processes, the thermal isomerization of xanthohumol to isoxanthohumol, which has better solubility but weaker activity, may occur [16,21]. Therefore, there is still a need to develop a method for isolating xanthohumol from hop extracts or post-extraction hop waste using a cheap, fast, and ecological method that can be used on an industrial scale. Additionally, there are possibilities for using waste products generated by the brewing industry, so-called hop waste, which is currently used as fertilizer in agriculture.

This work concerns the isolation of xanthohumol from hop residues remaining as a waste product after supercritical CO_2_ extraction by contacting the extract with magnetic iron oxide nanoparticles. Magnetic iron oxide nanoparticles (Fe_2_O_3_ or Fe_3_O_4_) have already been used for selective extraction [22]. Both unmodified nanoparticles, e.g., for the isolation of lutein and the removal of chlorophyll A from green plant extracts [23], as well as modified nanoparticles, e.g., with alizarin dyes [24], are used. A method for recovering metal ions from water or industrial waste using these nanoparticles has been described [11]. Magnetic iron oxide nanoparticles are safe from the point of view of human health and environmental protection. FDA has approved iron oxide nanoparticles for biomedical and clinical applications, in cell separation processes, as drug delivery vehicles [12], in magnetic resonance imaging [13], in anticancer therapy, and as a bone repair and tissue degradation agent [14].

In our previous work, the flavonoid-rich extract from spent hops was used for the synthesis of IONPs using a mixed method, i.e., chemical co-precipitation combined with biogenic surface modification [25]. In the next publication, we used iron oxide nanoparticles as a sorbent for the extraction of bitter acids (alpha- and beta-acids) from hop cone extracts in order to isolate xanthohumol [26]. The extraction conditions were optimized using the response surface methodology (RSM). The acetone extracts of hop cones used for the study were much richer in components compared to spent hop extracts, especially in beta acids, which are not present in the hop extract.

In this work, a waste product in the form of spent hops remaining after the supercritical CO_2_ extraction of hop cones was used. The elaborated procedure enables the management of a by-product generated in significant amounts by the brewing industry and its further use to obtain a valuable antioxidant, xanthohumol. The purification of the extract and isolation of xanthohumol and its recovery in a pure state can be utilized in such areas as the pharmaceutical, food, and cosmetic industries.

## 2. Materials and Methods

### 2.1. Synthesis of Iron Oxide NPs

Iron oxide NPs were synthesized by the co-precipitation method, as described previously [25,26,27]. Briefly, 0.1 M solutions of Fe(II) and Fe(III) salts were mixed in a molar ratio of 1:2. To maintain a pH in the range of 10–12, 25%, ammonia solution was added dropwise under constant stirring. The final mixture was separated into solid and liquid phases by an external magnetic field. The supernatant was discarded, and the separated precipitate was washed several times with deionized water to obtain pH 7. Then, the precipitates were air-dried for 24 h.

### 2.2. High-Performance Liquid Chromatography (HPLC) Analysis

HPLC analysis was performed using a Merck Hitachi LaChrom HPLC instrument (E. Merck, Darmstadt, Germany) equipped with a diode array detector, a column oven, and a solvent degasser. The analysis conditions were described in a previous work [26]. A column (150 mm × 4.6 mm I.D.) packed with 5 μm Zorbax Extend-C18 (pore size: 80 Å, surface area: 180 m^2^/g) from Agilent Technologies (Santa Clara, CA, USA) was thermostated at 25 °C ± 0.1. Retention data were recorded at a flow rate of 0.5 mL min^−1^ for 20% (0–3 min), 20–50% (3–6 min), 70% (6–15 min), and 100% (15–25 min) acetonitrile/water mobile phase containing 0.1% formic acid (*v*/*v*) [28]. Detection was set at 369 nm. The injection volume was 20 μL. Quantification of xanthohumol was performed based on a calibration curve in the range from 0.12 µg mL^−1^ to 160 µg mL^−1^.

### 2.3. Extraction of Plant Material

The extracted hops, a by-product of supercritical CO_2_ extraction, came from the Fertilizer Research Center of the Institute of New Chemical Syntheses of the Łukasiewicz Research Network in Puławy. Ultrasound-assisted extraction was carried out using 20 volume parts of ethyl acetate, propanol, acetone, 80% methanol, ethyl acetate-methanol (1:1, *v*/*v*), and propanol-methanol (1:1, *v*/*v*) [g/mL] per 1 part by weight of biomass. The extraction mixture was sonicated for 60 min in a Bandelin Sonorex RK 103 H ultrasonic bath (Bandelin Electronics, Berlin, Germany) with an ultrasonic power of 1200 W and a frequency of 35 kHz at a temperature below 20 °C. The extracts were filtered through Whatman No. 1 filter paper and stored in a refrigerator before further analysis.

### 2.4. Fourier Transform Infrared Attenuated Total Reflection (FT-IR/ATR) Spectroscopy

FT-IR/ATR spectra were recorded using a Nicolet 6700 spectrophotometer and a Harrick Meridian Diamond attenuated total reflection attachment. The spectra were recorded in the range of 4000–400 cm^−1^ at a resolution of 4 cm^−1^. Extracts were applied directly to the diamond crystal and left there until the solvent evaporated. The drying conditions used (room temperature) were essential to preventing the decomposition of xanthohumol. The interferograms comprised 256 scans, ensuring an optimal signal-to-noise ratio. The spectra were normalized by comparing the obtained spectrum to that of dried (48 h, 105 °C) spectrally pure potassium bromide. No smoothing functions were employed. Raw spectra were processed using OMNIC^TM^ software (Thermo Fisher Scientific Inc., Waltham, MA, USA) version 8.2.387.

### 2.5. Magnetic-Dispersive Solid-Phase Extraction (MSPE)

IONPs were added to the extract in the amount of 0.01 to 0.25 parts by weight per one volume part of the extract [g/mL]. In order to obtain satisfactory purification of the extract and to preserve as much xanthohumol as possible, in the case of the extract with ethyl acetate, 7–15 mg of IONPs was used per one volume part of the extract. For propanol, 15–50 mg of IONPs was used. For ethyl acetate-methanol (1:1, *v*/*v*) and propanol-methanol (1:1, *v*/*v*), 75–95 mg of IONPs was used. For acetone and 80% methanol extract, 30–40 mg and 250 mg of IONPs were added, respectively. The extraction mixtures were mixed in a rotator at 30 rpm at room temperature. After applying an external magnet, the liquid and solid phases were separated. The solid phase was removed, and the liquid phase was subjected to further analysis.

### 2.6. Conductivity Measurements

The kinetics of iron ion release from nanoparticles in the studied suspensions was monitored using a CX-401 multifunction meter (Elmetron, Zabrze, Poland) with a Euro EPS-2ZA sensor (POCH, Gliwice, Poland). IONPs obtained by co-precipitation were incubated for 48 h with different solvent systems. Changes in the conductivity values in µS of the mixtures were monitored over time. To ensure accurate conductivity measurements, the meter was calibrated using 80% methanol in distilled water (1.99 µS/cm) and 0.25 mM FeCl_3_ solution in 80% methanol (~146.28 µS/cm) at 25 °C. The procedure included zero-point adjustment with 80% methanol in distilled water and calibration with the FeCl_3_ solution.

The calibrated meter demonstrated linearity in the 0–146 µS/cm range with an R^2^ of 0.9753, suitable for low-conductivity measurements. The cell constant of the conductivity cell used in this work was 0.4 ± 0.05 cm^−1^. All measurements were temperature-controlled, and solvent samples were prepared to minimize moisture content, ensuring reliable results.

### 2.7. Dynamic Lights Scattering (DLS) Analysis

The Malvern Zetasizer Nano ZS analyzer (Malvern Instruments Ltd. Malvern Panalytical, Malvern, UK) was used to measure the average size of the particles at 25 °C.

### 2.8. Determination of Antioxidant Capacity

To investigate antioxidant capacity, SNAPC and FRAP tests were performed. All tests were performed in triplicate (*n* = 3) by measuring absorbance using a Genesys 20 spectrophotometer (ThermoSpectronic, Waltham, MA, USA) and Trolox (Sigma-Aldrich (St. Louis, MO, USA) as an antioxidant standard. To ensure the correctness of the determined antioxidant capacity, the extract concentration was adjusted by dilution/volume so that the measured absorbance was in the linear range of the appropriate calibration curves. The results were presented in mmol L^−1^ of Trolox per mL.

#### 2.8.1. Ferric Reducing Antioxidant Power (FRAP) Assay

The FRAP antioxidant activity of the extract before and after extraction was determined by a slightly modified version of the method described by Cañadas et al. [29], Oyaizu [30], and Brito et al. [31]. The FRAP working solution was a mixture of 300 mmol L^−1^ sodium acetate buffer (pH 3.6), 10 mmol L^−1^ TPTZ (2,4,6-tri-2-pyridinyl-1,3,5-triazine) in 40 mmol L^−1^ HCl, and 20 mmol L^−1^ FeCl_3_ in a 10:1:1 ratio. To generate the calibration curve, 3 mmol L^−1^ of aqueous/methanol (1:1, *v*/*v*) Trolox solution was diluted to 0.021875, 0.04375, 0.0875, 0.175, 0.35, and 0.7 mmol L^−1^. The linear regression equation of the absorbance versus concentration plot was as follows: (y = 1.215x + 0.1908; R^2^ = 0.9978). For the tests, 1450 µL of FRAP reagent and 50 µL of standard/extract/blank were mixed. After 15 min of incubation in the dark, the absorbance was measured at 593 nm.

#### 2.8.2. The Silver Nano Particle Antioxidant Capacity (SNPAC)

The SNPAC assay was based on the protocol previously developed by Özyürek et al. [32]. For the SNPAC assay, 2 mL of silver nanoparticles Ag-NP, x µL of sample/blank and (0.8 − x) mL of water were mixed. After 30 min of incubation at room temperature in the dark, the absorbance was measured at 423 nm. To generate a calibration curve, a series of Trolox solutions with concentrations of 3 mmol L^−1^ were added at different volumes from 0 to 100 µL. A calibration curve with the equation y = 0.0174x + 0.1709; R^2^ = 0.9879 was constructed based on the dependence of the observed increase in absorbance on the Trolox concentration. Silver nanoparticles (Ag-NPs) were prepared according to the previously described procedure [33]. Briefly, 260 mL of 1 mmol L^−1^ silver nitrate was mixed with 3 mL 1% aqueous solution of tripotassium citrate at 90 °C under constant stirring.

#### 2.8.3. DPPH Radical-Scavenging Assay

DPPH (1,1-diphenyl-2-picrylhydrazyl, Sigma-Aldrich, St. Louis, MO, USA) inhibition by the tested extracts/standard antioxidant was determined using the protocol of Brand-Williams et al. [34] with some modifications. Briefly, 20 µL of sample/standard was added to 1.5 mL of 100 mM DPPH solution (1.95 mg/50 mL methanol). The mixture was left in the dark at room temperature for 30 min. The decrease in absorbance was measured at 517 nm. The absorbance of the DPPH solution at 517 nm was 0.925 ± 0.032 (experimental control). Each sample was analyzed in triplicate. Data are given as the mean ± SD. The antioxidant activity of the sample was expressed as Trolox equivalent antioxidant capacity (TEAC) in micromoles of Trolox equivalents (TE) [35,36].

For the tested samples, TEAC (mM) was calculated using the linear regression equation (A = −0.5245 + 0.8465; R^2^ = 0.9903) obtained for a series of Trolox solutions in methanol: 0.05 mM; 0.1 mM; 0.2 mM; 0.4 mM; 0.8 mM; 1.6 mM [37].

### 2.9. Total Phenolic Content (TPC)

The total phenolic content (TPC) was evaluated using previously described protocols by Castaldo et al. [38], the Folin–Ciocalteau method [39,40], and de Camargo et al. [41], with a small modification. Briefly, 50 µL of sample/blank, 0.9 mL of deionized water, and 0.1 mL of the Folin–Ciocalteu reagent (Sigma-Aldrich, St. Louis, MO, USA) were mixed. After the addition of 1.0 mL of 10% Na_2_CO_3_, the mixture was kept in the dark for 2 h at room temperature. Then, the absorbance was measured at 760 nm. The data were recalculated into mg of gallic acid equivalent (GAE) per mL of sample using a calibration curve representing the GA concentrations 0.00390625, 0.0078125, 0.015625, 0.03125; 0.0625, and 0.125 mg mL^−1^ versus absorbance. The calibration curve equation was as follows: y = 6.9379x + 0.2234; R^2^ = 0.9901.

### 2.10. Total Flavonoid Content (TFC)

TFC was estimated according to the method of Fraisse et al. [42]. Briefly, after mixing 1.0 mL of 2% aluminum chloride (AlCl_3_) with 100 µL of sample/blank and 900 µL of water, the absorbance at 415 nm was measured after 30 min of incubation. The calibration curve represented the quercetin concentration (0.0625; 0.125; 0.25; 0.5; 0.75; 1.00 mg mL^−1^) versus absorbance. The TPC content of the extracts was converted to mg of quercetin equivalents (Q mg) per mL of extract.

### 2.11. Statistical Analysis

All measurements were performed in triplicate, and the results are expressed as the mean ± standard deviation (SD) or the percentage of the relative standard deviation (RSD%). The Kruskal–Wallis test was used for comparative analysis. The Kruskal–Wallis test, or one-way analysis of variance, is a nonparametric statistical test used to compare medians of independent data groups. In this case, the data did not meet the assumptions of normality and equal variance. Therefore, the Kruskal–Wallis test was used to compare independent data groups. The test was used to assess whether the distributions of values of individual data groups differed from each other, and the statistical significance was set at *p* > 0.05 [43]. To compare the DPPH test results before and after sorption in terms of similarity, the Mann–Whitney U test was used. Differences were considered statistically significant when *p* < 0.05. The statistical analysis was carried out using Microsoft Excel 2013 software and Statistica™ 13.3; 1984–2017 TIBCO Software Inc. (Palo Alto, CA, USA).

## 3. Results

### 3.1. Effect of Different Solvent Systems on the Recovery of Xanthohumol

#### 3.1.1. Aqueous-Alcoholic Solvent Systems

Methanol, ethanol, and alcohol–water extracts were mixed with different amounts of IONPs. The content of xanthohumol in the supernatant was determined by HPLC over time. The data summarized in Table 1 indicate that in 100% ethanol and 100% methanol, the extracts are very well purified, but these solvents do not provide sufficient recovery of xanthohumol (recovery < 10%). The addition of water improves the recovery of xanthohumol but weakens the sorption/elimination of other extract components. Therefore, to isolate pure xanthohumol, the addition of water to ethanol and methanol is not recommended. However, an exception is 80% methanol, which provides the most favorable conditions for obtaining a pure xanthohumol solution with a concentration of 43.23 µg mL^−1^, guaranteeing approximately 75% recovery of pure xanthohumol after 48 h of shaking 3 mL of extract with 750 mg of nanoparticles.

In order to illustrate the efficiency of purification of the extract enriched with xanthohumol, the total area of contaminating peaks was measured before and after incubation with IONPs at a wavelength of 280 nm. The peak areas together with the percentage of removed contaminants from the tested extracts are summarized in Tables S2 and S3. Figure S1 presents a chromatogram monitored at 280 nm of an extract prepared in 80% methanol before and after incubation with IONPs.

**Figure 1:**
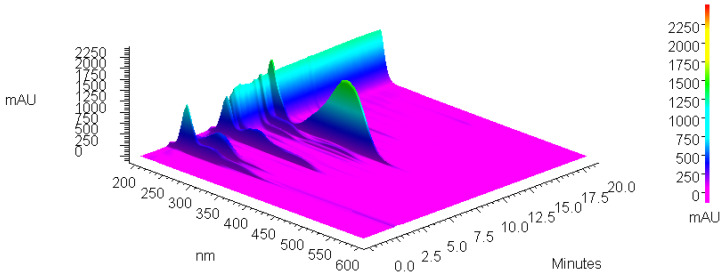
The 3D chromatogram of 80% methanol extract of spent hops.

**Figure 2:**
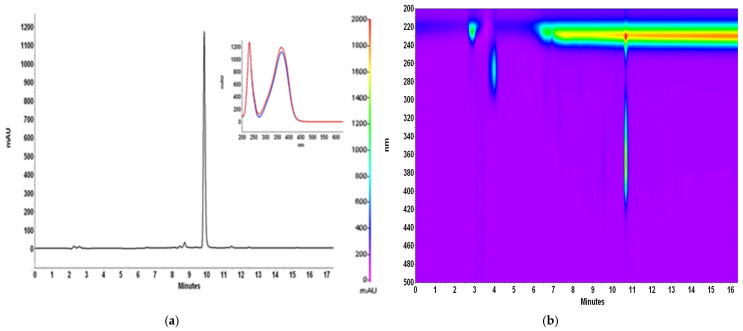
Chromatogram of 80% methanol extract of spent hop cones after 48 h of contact with 750 mg IONPs added, with insert representing the overlaid spectra of recorded peak (blue line) and xanthohumol standard (red line) (**a**); 2D chromatogram representing the relationship of time versus wavelength (**b**).

Figure 1 and Figure 2 show chromatograms of 80% methanol extract before and after IONP addition and UV–Vis spectra of isolated xanthohumol overlaid with the standard spectrum, which confirm its identity and degree of purity.

#### 3.1.2. Non-Aqueous Solvent Systems

Extracts from spent hop cones were prepared by ultrasound-assisted solvent extraction under the same conditions using different solvents: acetone, ethyl acetate, propanol, and mixed solvents, i.e., propanol-methanol (1:1, *v*/*v*) and ethyl acetate-methanol (1:1, *v*/*v*). The extract prepared in ethyl acetate should be purified using IONPs no later than 1 day after extraction due to xanthohumol degradation in this solvent.

**Figure 3:**
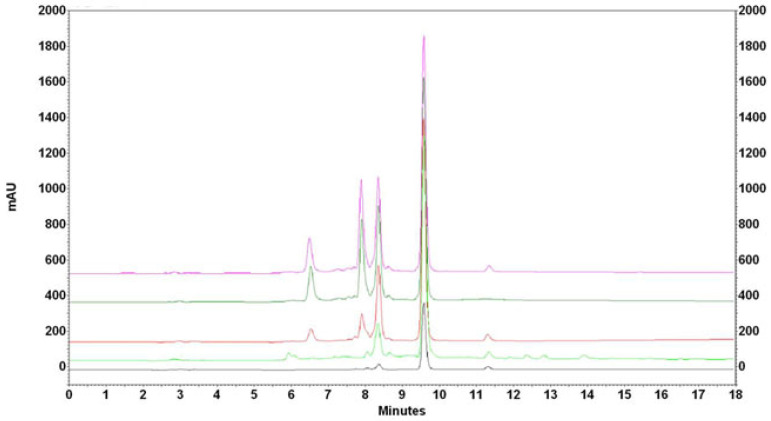
Overlaid chromatogram of acetone extract of spent hops (3 mL) with 100 mg IONPs recorded after 1 h, 2 h, 3 h, 4 h, and 5 h (from the top to the bottom).

The conditions for the purification of extracts from spent hop cones were optimized depending on the mass of added nanoparticles and the contact time of the extract with nanoparticles. Figure 3 shows overlaid chromatograms recorded every hour after mixing 3 mL of acetone extract with 100 mg of IONPs. As can be seen, the removal of impurities occurs in no less than 5 h. The extract before adding nanoparticles showed a xanthohumol content of 102.53 µg mL^−1^. After separating IONPs, the purified extract free of impurities showed a xanthohumol concentration of 14.26 µg mL^−1^, providing only 14% recovery.

**Figure 4:**
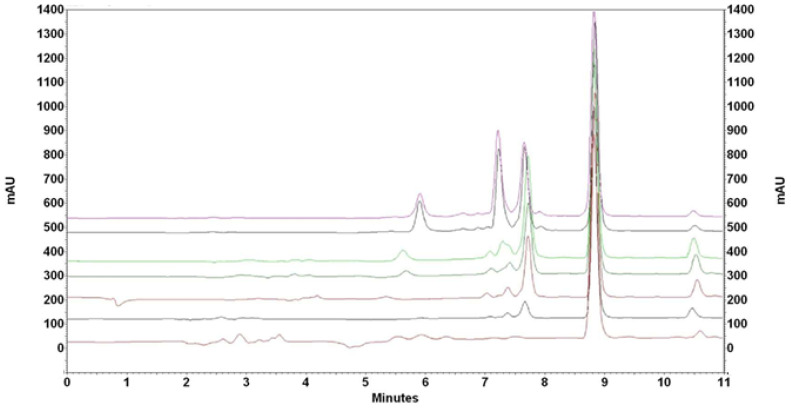
Chromatograms recorded after 5 h after mixing 1 mL of acetone extract of spent hops and 20, 40, 60, 80, 100, 110, and 130 mg of IONPs (from top to bottom).

The elimination of contaminants progresses not only with time but also with the increase in the mass of added nanoparticles. Figure 4 presents the overlaid chromatograms recorded 5 h after mixing 3 mL of acetone extract with an increasing mass of added nanoparticles.

**Figure 5:**
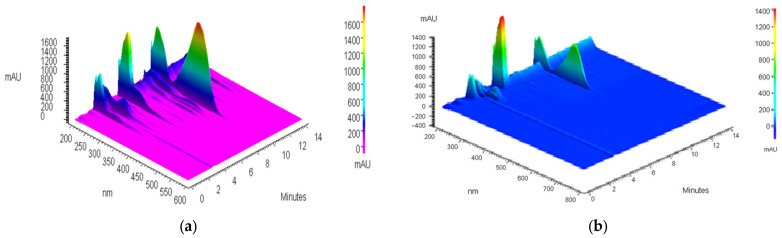
The 3D chromatograms of ethyl acetate-methanol extract (**a**) and extract recorded 3 h after mixing 1 mL of ethyl acetate-methanol extract of spent hops and 85 mg of IONPs (**b**).

The same method was used to optimize the purification conditions of extracts prepared in other solvents. The optimal conditions were determined on the basis of the experiments documented in the Supplementary Materials. The results and conclusions are summarized in Table 2. Among the solvents tested, the ethyl acetate-methanol mixture proved to be the most effective (Figure 5). The chromatogram of the extract and the extract purified with nanoparticles is shown in Figure 5. The chromatogram of the xanthohumol-rich solution shows, in addition to the xanthohumol peak, peaks eluted with the front, which are characteristic of the blank sample.

**Figure 6:**
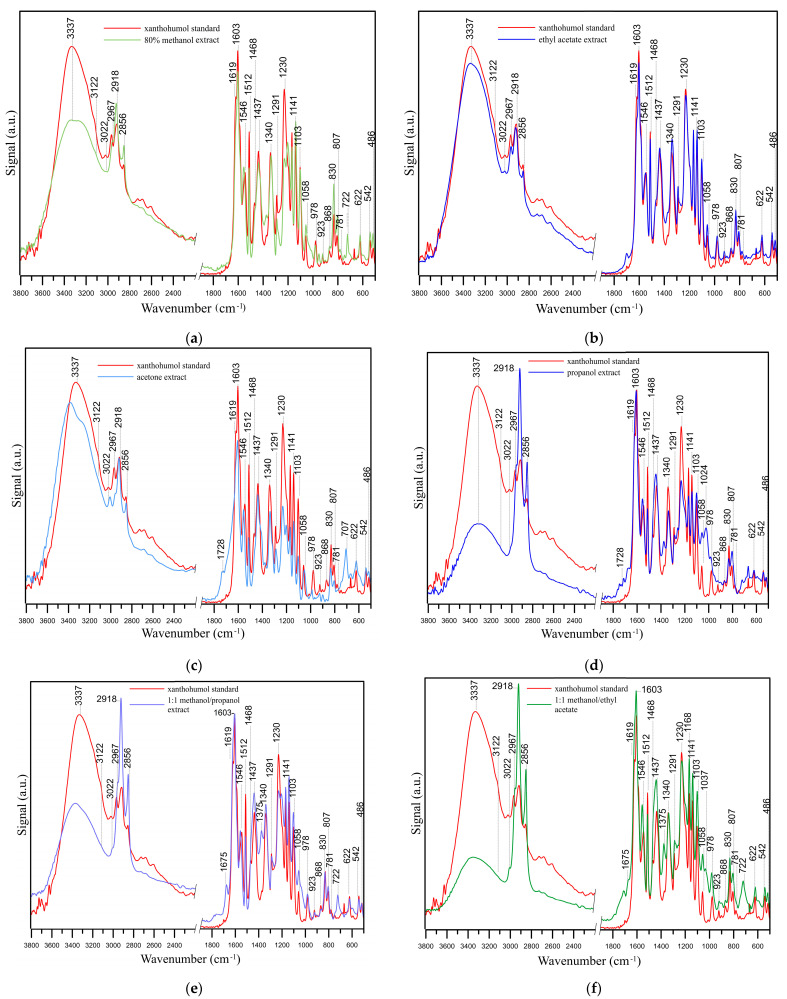
The FT-IR/ATR spectra of the purified extracts prepared in 80% methanol (**a**), ethyl acetate (**b**), acetone (**c**), propanol (**d**), and 1:1 mixtures of methanol-propanol (**e**) and methanol-ethyl acetate (**f**) compared to the xanthohumol standard.

Samples of purified extracts were dried at room temperature and analyzed by FT-IR/ATR. Figure 6a–f shows the IR spectra of the purified extracts. In each figure, the spectrum of the particular extract was compared with the spectrum of the standard xanthohumol. The standard spectrum of xanthohumol exhibits characteristic bands, including a broad band of –OH groups, connected by inter- and intramolecular hydrogen bonds at 3337 cm^−1^, and C-H stretching vibrations in the aromatic ring at 3122 cm^−1^ and within the 923–781 cm^−1^ and 622–486 cm^−1^ ranges. Vibrations at 3022 cm^−1^ and in the range of 1603–1512 cm^−1^, as well as at 978 cm^−1^, are indicative of the presence of -C=C- groups. The bands of –CH_3_ groups vibrations can be observed in the ranges of 2967–2856 cm^−1^ and 1468–1373 cm^−1^ (C-H bending vibrations). The spectrum also displays a C=O stretching vibration band at 1619 cm^−1^, a band of –OH group bending vibrations in the range of 1340–1291 cm^−1^, a band of C-O group vibrations at 1230, 1141, 1103, and 1058 cm^−1^, and coupled vibration bands of C–O and O–H at 1169 cm^−1^ [44,45,46,47]. The position and intensity of the bands of purified xanthohumol isolated with different solvents or solvent mixtures are practically identical to those in the IR spectrum of the xanthohumol standard, thereby confirming that these are the same compounds. The presence of bands at 1728, 1675, and ~722 cm^−1^ (Figure 6a–f) may be indicative of incomplete solvent evaporation while drying the samples on the ATR crystal. The drying conditions employed (room temperature), which are requisite in this instance in order to prevent xanthohumol decomposition, may exert an influence on the presence of solvent residues in the tested extracts. Furthermore, the presence of solvents can be discerned in the range of C-H group vibrations, as evidenced by the higher intensities of bands within the 2967–2856 cm^−1^ and 1468–1373 cm^−1^ ranges. The appearance of additional bands in the spectra of extracts within the 1800–1700 cm^−1^ range may also indicate the presence of minor quantities of compounds containing C=O groups (carboxylic acids, aldehydes, ketones) in the extracts. Most likely, they come from beta acids, which are notoriously challenging to remove. The bands responsible for the presence of C=O groups are typically among the most intense in IR spectra. Consequently, even trace amounts of compounds containing C=O groups in their structure will be discernible in IR spectra.

### 3.2. Characterization of IONPs Before and After Sorption

**Figure 7:**
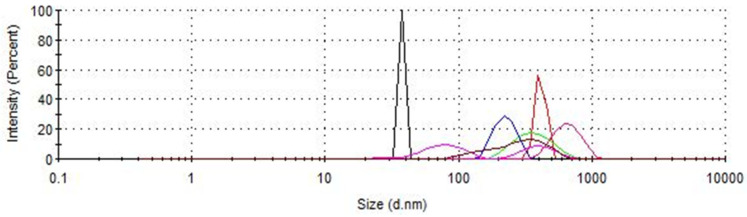
DLS measurement for synthesized IONPs before (red line) and after purification of spent hops extracted using different solvent systems: 80% methanol (blue line), ethyl acetate (brown line), acetone (green line), propanol (black line), 1:1, *v*/*v* ethyl acetate-methanol (pink line), 1:1, *v*/*v* propanol-methanol (deep violet line).

**Figure 8:**
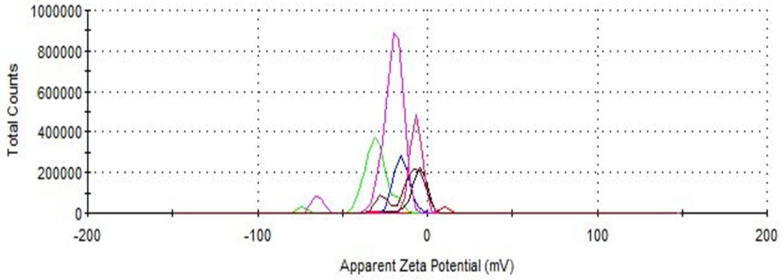
Zeta potential measurement for synthesized IONPs before (red line) and after purification of spent hops extracts. The colors of the lines are the following: 80% methanol (green line), ethyl acetate (deep violet line), acetone (blue line), propanol (black line), 1:1, *v*/*v* ethyl acetate-methanol (brown line), 1:1, *v*/*v* propanol-methanol (pink line).

The hydrodynamic diameters of the synthesized IONPs before and after extract purification were studied by light scattering. As shown in Figure 7, the hydrodynamic diameter of the synthesized IONPs is 414 nm, but after their use for extract purification, it decreases, with the smallest value for propanol (37.84 nm), regardless of the solvent system used, except for the propanol-methanol extract, where the diameter increases to 663.5 nm. The zeta potential analyzer was used to measure the surface charge of the IONPs. While the synthesized nanoparticles have a positive zeta potential of +10.2 mV (Figure 8), after extraction, the zeta potential generally decreases. It can be seen that the synthesized nanoparticles in aqueous solution form two populations with different surface charges, but the positively charged population is dominant. After the purification process of the extracts, the nanoparticles clearly change their surface charge to negative from −4.78 mV to −29.9 mV, which indicates the sorption of charged extract components. The smaller population of nanoparticles used reaches even more negative values, up to −94.3 mV in the case of nanoparticles incubated in ethyl acetate extract. The polydispersity index (PDI) ranges from 0.259 for nanoparticles incubated in 80% methanol to 1 for a set of solvents containing propanol and ethyl acetate-methanol, which indicates a high degree of polydispersity. The DLS measurement parameters are summarized in Table 3.

### 3.3. Characterization of Antioxidant Capacity of Extracts Before and After Sorption onto IONPs

The antioxidant capacity of six spent hop extracts obtained in different solvents was assessed using SNAPC and FRAP methodologies. FRAP assesses the ability of the sample to reduce Fe^3+^ ions to Fe^2+^, while SNAPC assesses the ability to reduce silver ions. The antioxidant capacity in both cases was converted to µM Trolox equivalents per mL of extracts. As summarized in Table 4, the highest antioxidant capacity was found in acetone and 80% methanol extracts. The 80% methanol extract showed strong results in the FRAP (0.4770 ± 0.01 µmol Trolox/mL), SNAPC (3322.41 ± 0.01 µmol Trolox/mL), and DPPH (1.554 mM Trolox/mL) assays. Extracts prepared in propanol and ethyl acetate had the lowest antioxidant activity. The mixed solvents methanol-ethyl acetate and methanol-propanol did not differ significantly in terms of antioxidant capacity. The antioxidant capacity expressed as Trolox equivalent in chosen tests showed a similar trend for total phenolic content (TPC) as well as total flavonoid content (TFC). Phenolic compounds are considered the main components of hop cone extracts, with xanthohumol being the main polyphenol belonging to the flavonoid family. The TPC data showed a positive correlation with the corresponding SNAPC and FRAP data with R^2^_TPC/FRAP_ = 0.9880; R^2^_TPC/SNAPC_ = 0.8479, similar to the TFC data, which correlated with SNAPC and FRAP, with R^2^ values of 0.6210 and 0.8762, respectively. In the case of the DPPH test, these correlations are the weakest. Both the FRAP and SNAPC tests assess the total reducing capacity of the extracts. Due to their transparent mechanism and stable kinetics in the reaction with Trolox as a standard antioxidant, they provide precise results with low variability [48]. The RSD% of the measurements did not exceed 7%, except for two single exceptions concerning ethyl acetate and propanol extracts, probably because these solvents do not provide sufficient miscibility with aqueous additives. It should be noted that after adding IONP to the extracts, the measured antioxidant tests showed lower values of all parameters considered, except for the TFC recorded for the ethyl acetate-methanol extract, indicating that part of the xanthohumol content together with other extract components contributing to the total antioxidant capacity were removed. This is clear evidence of the usefulness of IONP as an effective sorbent. The DPPH test before and after sorption did not show any significant changes in the antioxidant activity of the extracts (*p* = 0.390; Mann–Whitney U test). The DPPH test should not be recommended for testing the antioxidant activity of extracts after purification by magnetic dispersive extraction using IONP. Nanoparticles themselves act as additional antioxidants in this test. At 100 mM DPPH, the IC50 for IONP is 65 mg/mL. IONPs can quench DPPH free radicals in a dose-dependent manner, which is probably due to the neutralization of the free radical by electron transfer. However, the activity of IONPs depends on the size of the nanoparticles [49],; therefore, the fraction of the smallest IONPs remaining in the supernatant may show higher activity than the purified extract itself, hence the lack of noticeable changes in the results of the DPPH test before and after sorption were observed.

The results of antioxidant tests (except DPPH) determined for individual extracts prepared using different solvents were tested using the Kruskal–Wallis test. The test result confirms that the type of solvent has a significant effect on antioxidant activity (*p* < 0.05; Kruskal–Wallis test). This difference is due to the different quantitative and qualitative composition of the extracts [50].

### 3.4. Kinetics of Iron Ions Release from IONPs in Aqueous Suspensions

**Figure 9:**
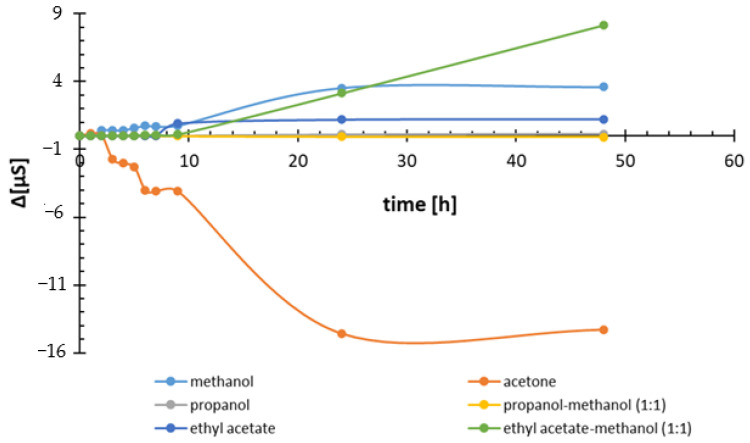
Conductivity changes (µS) of IONP suspension prepared in different solvent systems at optimized concentration (Table 1 and Table 2) as a function of time (h).

It is known that there is a possibility of releasing metal ions from nanoparticles into the dispersion medium. An example is Ag-NP nanoparticles, which show toxicity, among other reasons, due to the release of Ag^+^ ions [33,51]. To check the possibility of contamination of the purified xanthohumol extract by the release of ions from IONPs, the conductivity value of the mixtures was monitored over time. Iron ions have a fairly high value of the limiting molar conductivity Λ_0_ 1/2Fe^2+^ = 54 × 10^−4^ m^2^ S mol^−1^ and 68 × 10^−4^ m^2^ S mol^−1^ for 1/3 Fe^3+^ [52], thanks to which their release affects the conductivity measurement. Figure 9 shows the change in conductivity of the studied dispersion containing optimized IONP concentration converted to 10 mL of the extraction mixture volume as a function of time.

Incubation of IONPs in different solvents for optimized time periods, i.e., 24 h for 80% MeOH, 5 h for acetone-propanol, 3 h for propanol-methanol and ethyl acetate-methanol, and 1 h for ethyl acetate, causes a slight increase in conductivity from 0.21 to 4.68%, except for the mixture of IONPs in acetone and propanol-methanol, where decreases in conductivity of 6.59 and 0.05% are observed, respectively.

## 4. Discussion

In this work, an effective ultrasonic-assisted solvent extraction was used, which uses ultrasound to generate cavitation, supporting the process of diffusion of active compounds into the solvent. The obtained 80% MeOH extract can be purified using the addition of IONPs at room temperature for 48 h. The final product contains 74.61% of the initial xanthohumol content. When recalculated on the basis of the mass of the biomass, it is 865 µg/g of spent hops. This xanthohumol recovery is smaller than that using Grudniewska and Popłoński’s [15] method; however, our proposition does not require many steps and expensive reagents. Due to the magnetic properties of IONPs, there is no need for additional time-consuming steps such as filtration or centrifugation during separation processes. Commonly known disadvantages of IONPs are problems with agglomeration and stability and susceptibility to oxidation. However, in the case of dispersive extraction, especially when we only sorb the contaminants we want to remove, these disadvantages do not have any significance for the efficiency of the process.

Regarding the hydrodynamic diameter and zeta potential of nanoparticles, sorption of extract components results in a decrease in both surface charge and size. This is a beneficial phenomenon, as a negative zeta value ensures good dispersion stability in an aqueous environment [53]. A similar role is played by size reduction. Hydrophilic unmodified nanoparticles have a larger hydrodynamic diameter, while blocked hydroxyl groups in the adsorption process cause a decrease in hydrophilicity and aggregation, leading to a decrease in hydrodynamic size [54]. The changes observed in the size and surface charge of nanoparticles after sorption are the result of retaining extract components on the active centers of the IONP surface. The adsorption sites of IONPs are hydroxyl groups and coordinatively unsaturated Fe^3+^ and Fe^2+^ ions, which have Lewis acid properties and are thus able to coordinate molecules containing free electron pairs. The adsorption process can be mediated via electrostatic, donor–acceptor, and hydrophobic interactions and/or hydrogen bonds [55].

In our study, the antioxidant activity experiments, similar to HPLC analysis, confirm the decrease of polyphenols content after treatment with IONPs. Sorption of polyphenolic compounds protects against the prooxidative activity of bare IONPs. This aspect was investigated through autoxidation experiments of styrene in the work of Scurti et al. [56]. IONPs, a material containing Fe^2+^ ions on the surface, have the ability to participate in the heterogeneous Fenton reaction, which results in its prooxidant abilities. However, experiments with inhibited autoxidation show that IONPs modified with phenolic compounds lose their antioxidant activity. This result is due to the fact that phenols have the ability to bind to the iron oxide surface using phenolic functional groups, which prevents them from participating in the reaction with a free radical by hydrogen transfer. The authors of the study also suggest that the IONP–phenol bond provides stability even in an oxidizing environment.

A limitation of this study is the possible content of iron in the extract, which may cause deterioration in the quality of the tested extracts due to oxidation reactions. However, we have no control over the iron content because its source is the raw material [57]. The conducted studies only confirm that iron is not released during the optimized incubation time from the added nanoparticles, which, after synthesis, were subjected to several washings from excess ions. However, longer incubation is not recommended due to the possibility of ion release. Conductivity measurements confirmed this possibility, especially in the case of a solvent system containing methanol and water. In contrast to the electrical conductivity of electrolytes in water, conductivity in organic solvents is much less well-documented [58]. Studies on electrical conductivity in non-aqueous solutions were conducted at the turn of the 20th century by Kahlenberg and Lincoln [59,60]. Pioneering studies showed that electrical conductivity in non-aqueous solvents increases with the increase in the dielectric constant of the medium, indicating the participation of dissociation and ionic conductivity in addition to molecular conductivity. A small increase in conductivity over time measured during the incubation of IONPs in different solvents indicates an increase in the concentration of ionic substances. The greatest increases in conductivity were noted for solvents with the highest dielectric constant, i.e., systems with water (ε = 78.39) and methanol (ε = 32.6). An exception is acetone, where despite the high dielectric constant (ε = 20.7), conductivity decreased during incubation. The reason for this atypical behavior may be phenomena of an opposite nature, reducing the number of ions as a result of ion association into ion pair complexes, which clearly reduced the number of free ions in the solution and ion diffusion. Such phenomena have already been observed in solutions in polyethylene glycol [61] and for room-temperature ionic liquids in acetone [62].

Another limitation of our study is the lack of quantitative and qualitative identification of the remaining extract components. To carry out these studies, it would be necessary to use mass spectrometry detection and have standards of individual components to make calibration curves. The results of previous studies have shown that extracts from used hop cones are rich in polyphenolic compounds, of which over 30 were identified, including flavanols (derivatives of quercetin and kaempferol), hydroxycinnamic acids (neochlorogenic acid, chlorogenic acid, cryptochlorogenic acid, feruloylquinic acid), proanthocyanidin oligomers, flavan-3-ol monomers, dimers and trimers (catechin, epicatechin), and flavonol glycosides [63,64]. Our study aimed to efficiently remove as many contaminants as possible and to quantitatively determine the desired component, which was xanthohumol.

## 5. Conclusions

A new, cheap, ecological, and simple method of purifying extracts from spent hop cones using IONPs was proposed to recover xanthohumol, one of the most powerful antioxidants. The use of magnetic IONPs as a sorbent in optimized incubation conditions, most preferably in 80% MeOH and or ethyl acetate-methanol (1:1, *v*/*v*), allows for the purification of the extract without the need for laborious preparative isolation procedures. The final product is a solution of pure xanthohumol with a concentration of 43 µg mL^−1^, which eliminates the need for the desorption step, usually necessary in liquid–solid extraction. The extract purification procedure is cheap and allows for the use of waste products from the brewing industry as sources of xanthohumol.

## 6. Patents

Polish patent (P.450206, 5 November 2024): “Method of isolating xanthohumol from spent hop extracts (*Humulus lupulus* L.)”.

## Figures and Tables

**Table 1:** Effect of water addition to methanol and ethanol on xanthohumol recovery from 3 mL spent hop extract. Quantitative measurements were performed using the HPLC method according to the conditions described in Section 2.2. The peak areas of xanthohumol before and after incubation of individual extracts with IONPs are summarized in Table S1.

Solvent Kind	Before IONP Addition Conc. [µg mL^−1^]	After IONP Addition Conc. [µg mL^−1^]	Recovery [%]	Impurities [+/−]	IONP Mass [mg/3 mL Extract]	Contact Time [h]
MeOH	108.25 ± 0.43	8.33 ± 0.17	7.69	-	350	24
80% MeOH	57.94 ± 0.56	43.23 ± 0.52	74.61	-	750	48
50% MeOH	66.82 ± 0.43	36.34 ± 0.38	54.38	+	350	24
EtOH	88.89 ± 1.49	8.33 ± 0.5	9.37	-	350	24
80% EtOH	108.43 ± 1.43	70.84 ± 0.54	65.33	+	700	48
50% EtOH	96.25 ± 0.63	84.83 ± 0.78	88.13	+	350	24

**Table 2:** Effect of non-aqueous solvents on xanthohumol recovery from 1 mL spent hop extract.

Solvent System	Before IONP Addition Conc. [µg mL^−1^]	After IONP Addition Conc. [µg mL^−1^]	Recovery [%]	IONP Mass [mg]	Contact Time [h]
propanol	90.44 ± 1.12	33.63 ± 0.50	37.18	17 (15–50)	5.5 (5–5.7)
ethyl acetate	57.71 ± 0.75	23.43 ± 0.62	40.61	10 (7–15)	0.8 (0.5–1)
acetone	102.53 ± 0.41	14.26 ± 0.40	13.91	33 (33–43)	5 (≥5)
propanol-methanol	82.02 ± 0.51	21.84 ± 0.16	26.63	85 (75–95)	3 (1–4)
ethyl acetate-methanol	77.06 ± 0.61	36.09 ± 0.16	46.83	85 (75–95)	3 (1–4)

**Table 3:** DLS measurement parameters obtained for aqueous mixture of IONPs before and after purification procedure of spent hops in different solvent systems.

Measurements (Intensity)													
Sample Name	Intensity PSD Hydrodynamic Diameter [nm]				Volume PSD [nm]				PDI	Zeta Potential [mv]			
	Peak 1	% Intesity	Peak 2	% Intesity	Peak 1	% Volume	Peak 2	% Volume		Peak 1	Area [%]	Peak 2	Area [%]
IONPs	414.2	100	\	\	420.1	100	\	\	0.838	10.2	79.1	−28.8	20.9
80%MeOH	372.8	100	\	\	391.4	100	\	\	0.259	−29.9	96.7	−73.7	3.3
acetone	225	100	\	\	191.8	100	\	\	0.396	−15.4	100	/	/
propanol	37.84	100	\	\	38.04	100	\	\	1	−4.78	98	−23.1	2
propanol-MeOH	663.5	100	\	\	716.9	100	\	\	0.451	−6.7	100	/	/
ethyl acetate	78.62	57.8	410.2	42.2	26.5	31.5	59.22	67.4	0.391	−19.7	94.3	−94.3	5.7
ethyl acetate-MeOH	75.23	100	\	\	75.23	100	\	\	1	−7.73	80.1	−26.8	19.9

**Table 4:** Antioxidant capacity and TPC of spent hops extracts in different solvents before and after sorption onto IONPs. Data are presented as mean ± SD.

Extract	Before Sorption onto IONPs									After Sorption onto IONPs								
	µM ^1^/mM ^2^ Trolox/mL					mgGAE/mL		mg Q/mL		µM ^1^/mM ^2^ Trolox/mL					mg GAE/mL		mg Q/mL	
	FRAP ^1^	RSD %	SNAPC ^2^	DPPH ^2^	RSD %	TPC	RSD%	TFC	RSD %	FRAP ^1^	RSD%	SNAPC ^2^	DPPH ^2^	RSD %	TPC	RSD %	TFC	RSD %
80%MeOH	0.477	0.09	3.322	1.554	3.48	0.084	0.09	0.383	0.08	0.119	0.00	1.293	1.548	3.36	0.032	0.45	0.305	0.38
acetone	0.156	0.81	0.989	0.467	0.48	0.045	0.00	0.276	0.00	−0.033	−2.88	0.368	0.471	0.10	−0.006	−2.25	0.141	0.42
propanol	−0.049	−0.96	0.701	0.070	0.24	0.010	2.18	0.063	0.49	−0.075	−0.64	0.299	−0.018	0.12	−0.027	−27.97	0.034	7.79
ethyl acetate	−0.096	−2.74	0.552	−0.101	0.12	0.002	12.80	0.054	2.27	−0.139	−0.34	0.345	−0.103	0.06	−0.022	−0.77	0.028	6.19
propanol-MeOH	0.089	1.07	0.902	0.652	0.44	0.032	0.26	0.253	0.32	−0.047	−1.00	0.604	0.535	0.00	−0.005	0.00	0.205	1.29
ethyl acetate-MeOH	0.071	0.67	0.891	0.675	0.22	0.023	0.62	0.176	0.63	−0.035	−1.72	0.351	0.618	0.22	−0.006	−2.30	0.195	1.07

**Abbreviations**: ^1^ the values of antioxidant activity expressed in µM of the standard antioxidant, ^2^ the values of antioxidant activity expressed in mM of the appropriate reference standard; TPC: total polyphenol content; antioxidant activity tests: FRAP: iron-reducing antioxidant capacity; GAE: gallic acid equivalent; Q: quercetin; SNPAC: antioxidant capacity of silver nanoparticles. To estimate the antioxidant activity of the extracts tested by the SNAPC method, six different volumes of extracts from 0 to 100 µL were added to the silver nanoparticles seed solution. Increasing the volume of the extract resulted in an increase in absorbance, even beyond the absorbance of 1 (80% methanol), and in the case of propanol and ethyl acetate extracts, there was a loss of linearity above 80 µL of added extract. Therefore, in order to compare the antioxidant capacity and convert to Trolox equivalent for different extracts, the absorbance measured for a volume of 30 µL was selected.

## Data Availability

The dataset is available upon request from the authors.

## Supplementary Materials

The following supporting information can be downloaded at: https://www.mdpi.com/article/10.3390/antiox14030314/s1, Figure S1: Chromatogram of an extract prepared in 80% methanol before (top chromatogram monitored at 280 nm) and after (bottom chromatogram monitored at 369 nm) incubation with nanoparticles; Table S1: Effect of water addition to methanol and ethanol on xanthohumol recovery from 3 mL spent hop extract. Quantitative measurements were performed using the HPLC method according to the conditions described in Section 2.2. Detection was set at 369 nm; Table S2: Effect of non-aqueous solvents on xanthohumol recovery from 1 mL spent hop extract. Detection was set at 369 nm; Table S3: Effect of solvents on the percentage of contaminant removal. The total areas of the contaminating peaks of xanthohumol extract were recorded at 280 nm. The conditions of incubation of extracts with nanoparticles were the same as those described in Tables S1 and S2 for the individual solvent systems.
