# Supplementary material for: Purification of Spent Hop Cone (Humulus lupulus L.) Extract with Xanthohumol Using Mesoporous Superparamagnetic Iron Oxide Nanoparticles
Source: Antioxidants (Basel). 2025 Mar 5;14(3):314. doi: 10.3390/antiox14030314 (PMC11939198; doi:10.3390/antiox14030314)
Supplement: Supplementary file 1 [file antioxidants-14-00314-s001.zip › antioxidants-3369689-supplementary.pdf]

Table S1. Effect of water addition to methanol and ethanol on xanthohumol recovery from 3 mL spent hop extract. Quantitative measurements were performed using the HPLC method according to the conditions described in subchapter 2.2. Detection was set at 369 nm.

solvent kind	Before IONPs addition Peak'area	After IONPs addition Peak'area
MeOH	82626236	6771643
80% MeOH	44438520	33269690
50% MeOH	51176271	28034190
EtOH	67931089	6771643
80% EtOH	82765679	54228199
50% EtOH	73521082	64847329

Table S2. Effect of non-aqueous solvents on xanthohumol recovery from 1 mL spent hop extract. Detection was set at 369 nm.

solvent system	Before IONPs addition peak area	After IONPs addition peak area
Propanol (PrOH)	69110728	25979326
ethyl acetate	44260694	18241430
acetone	78285430	11275905
PrOH-MeOH	62717660	17032512
ethyl acetate-MeOH	58947514	278466020

Table S3. Effect of solvents on the percentage of contaminant removal. The total areas of the contaminating peaks of xanthohumol extract were recorded at 280 nm. The conditions of incubation of extracts with nanoparticles were the same as those described in Table S1 and S2 for the individual solvent systems.

solvent system	Σ peak area of impurities		Removal [%]
	Before incubation with IONPs	After incubation with IONPs	
100%MeOH	141318412	0	100
80%MeOH	197342295	0	100
50% MeOH	185128383	8401794	95.46
100% EtOH	103716210	0	100
80% EtOH	250809554	34543161	86.23
50% EtOH	141670586	52139383	63.20
ACETON	81612821	0	100
PrOH	15561212	0	100
Ethyl acetate	18746777	1121710	94.02
PrOH+MeOH	65163462	0	100
Ethyl acetate+MeOH	52413917	0	100

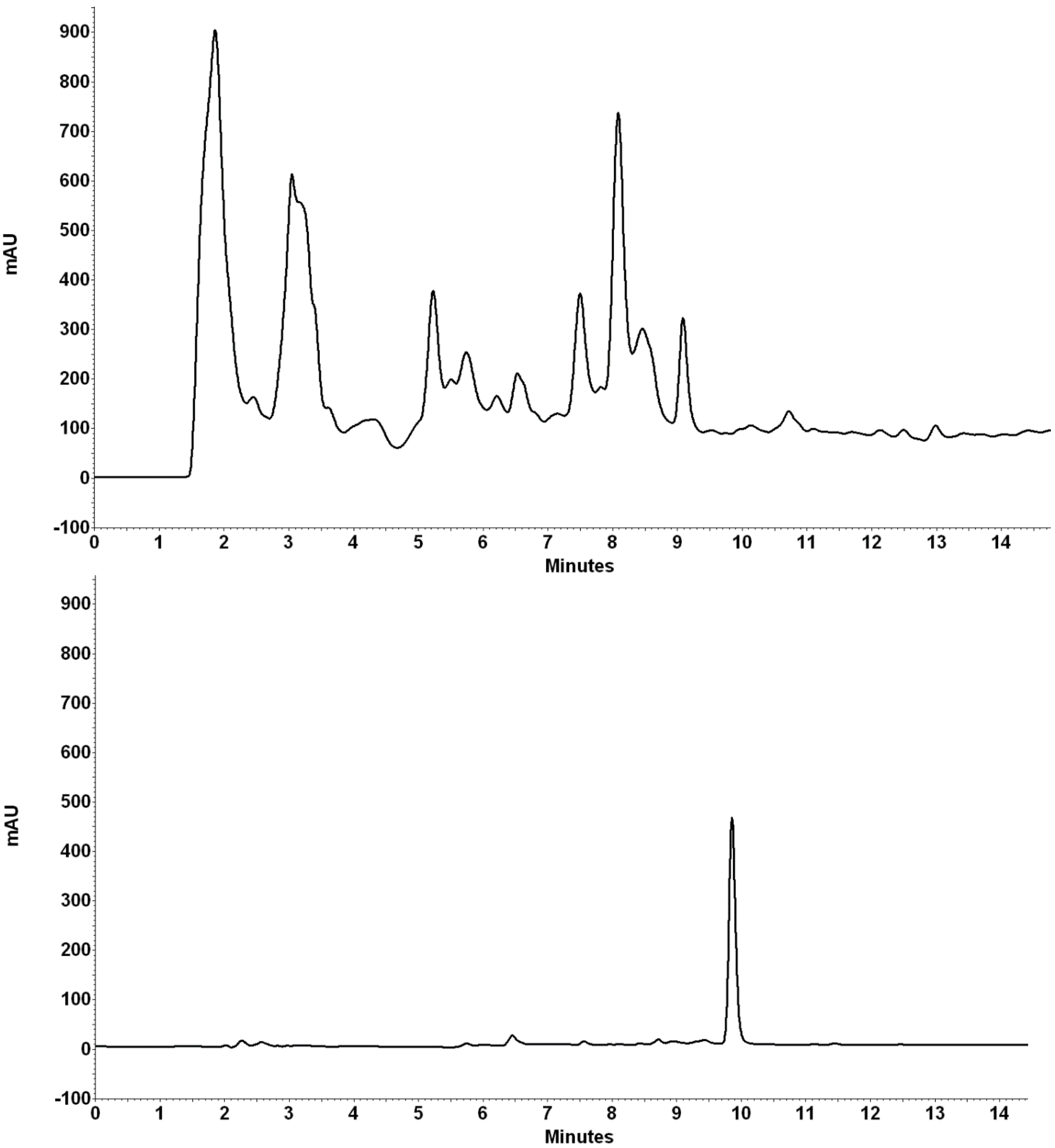

Figure S1. Chromatogram of an extract prepared in 80% methanol before (top chromatogram monitored at 280 nm) and after (bottom chromatogram monitored at 369 nm) incubation with nanoparticles.
